# Comparative effectiveness research on proximal femoral nail versus dynamic hip screw in patients with trochanteric fractures: a systematic review and meta-analysis of randomized trials

**DOI:** 10.1186/s13018-022-03189-z

**Published:** 2022-06-03

**Authors:** Hong Xu, Yang Liu, Erdem Aras Sezgin, Šarūnas Tarasevičius, Robin Christensen, Deepak Bushan Raina, Magnus Tägil, Lars Lidgren

**Affiliations:** 1 Department of Orthopaedics, Xiaoshan Traditional Chinese Medical Hospital, Hangzhou, Zhejiang Province China; 2 Department of Clinical Sciences Lund, The Faculty of Medicine, Orthopedics, Lund University, Lund, Sweden; 3 Department of Orthopaedics&Traumatology, Faculty of Medicine, Aksaray University, Aksaray, Turkey; 4 Department of Orthopedics, Lithuanian University of Health Sciences, Kaunas, Lithuania; 5 Section for Biostatistics and Evidence-Based Research, The Parker Institute, Bispebjerg and Frederiksberg Hospital, Copenhagen, Denmark; 6 Research Unit of Rheumatology, Department of Clinical Research, Odense University Hospital, University of Southern Denmark, Odense, Denmark

**Keywords:** Trochanteric fractures, Dynamic hip screw, Proximal femur screw, Meta-Analysis, Implant failure, Revision surgery

## Abstract

**Background:**

The treatments for trochanteric fractures try to regain early mobility and limit morbidity and risk of reoperations. The most currently used dynamic hip screw (DHS) and the proximal femoral nail (PFN) are both with pros and cons. We aimed to assess the comparative effectiveness of these interventions for trochanteric fractures by evaluating the surgical performance and postoperative outcomes.

**Methods:**

PubMed, Web of Science and Cochrane Central Register were searched for RCTs comparing DHS and PFN for trochanteric fractures. All selected studies and the risk of bias were assessed. Clinical data including operative time, intraoperative blood loss, intraoperative fluoroscopy time, successful closed reduction and complications like nonunion, implant failure and reoperation were recorded. Random-effects models were used in Review Manager software, and GRADE was applied for the interpretation of the evidence.

**Results:**

From 286 identified trials, twelve RCTs including 1889 patients were eligible for inclusion; six RCTs directly comparing DHS with PFN, while other six compared DHS with proximal femoral nail antirotation (PFNA). Compared to DHS, PFN had shorter operative time and led to less intraoperative blood loss. However, DHS need less intraoperative fluoroscopy time than PFN. No difference was seen for the achievement of closed reduction. For risk of postoperative complications, no difference was seen between PFN and DHS for non-union, risk of implant failure and revision surgery.

**Conclusions:**

PFN(A) resulted in a shorter operative time and less intraoperative blood loss compared to DHS. However, no difference was seen for postoperative complications.

*Trial registration* PROSPERO: CRD42021239974.

**Supplementary Information:**

The online version contains supplementary material available at 10.1186/s13018-022-03189-z.

## Background

Hip fractures are a major burden to both the individuals and society, leading to disability or even mortality for the elderly patients and cause huge economic cost [1, 2]. As the number of elderly people is increasing world-wide, it has been estimated that the number of hip fractures will rise to 2.6 million by 2025, and to 6.25 million in 2050 [3]. Trochanteric fractures comprise approximately 50% of the hip fractures and are often caused by a low-energy fall [4]. The trochanteric bone often retains a good vascular supply after fracture, with a high union rate compared to femoral neck fractures [5, 6]. However, the mortality after trochanteric fractures still ranges from 12 to 41% within the first 6 months [7].

Different devices have been used for the fixation of trochanteric femoral fractures with the following two being the most commonly used: dynamic hip screw (DHS) and proximal femoral nail (PFN). DHS, introduced in the 1970s, could provide both the dynamic and static support to stabilize the fracture. However, complications related to screw displacement are not uncommon such as distal extrusion of the screw and secondary fracture displacement [8]. The PFN was developed by the AO/ASIF in 1996 with an intramedullary device conceptualized as a less invasive alternative especially for the treatment of unstable trochanteric and subtrochanteric femoral fractures [9]. In 2003, the proximal femoral nail antirotation (PFNA) system was introduced with a helically shaped sliding column-blade design, providing an increased contact-area between bone and implant preventing the rotation induced cut-outs [10, 11]. An intramedullary device has some theoretical advantages over extra-medullary devices by-passing the need of fix the plate to the shaft with screws, which can be difficult in osteoporotic bones. In addition, shaft fixation in PFN is closer to the center of rotation of the hip. The load is thereby transmitted to the femur, along a more medial axis, which results in a shorter level arm [12]. Nowadays, PFN device has been used widely in the clinic and provided by different brands with various length, diameter, neck shaft angle, number of cephalic screws, ability to control rotation and construction materials [13]. Even though PFN has more theoretical benefit than DHS, there is still ongoing controversy whether PFN is a better choice than DHS in the literature especially from clinical studies. In recent large registry studies, Grønhaug KML et al. showed that PFN is only suggested for unstable trochanteric and subtrochanteric fractures, but not for stable fractures or individual fracture types [14]. Wolf O et al. showed that a slightly increased risk of death up to 30 days postoperatively was seen for patients under PFN compared to DHS in stable trochanteric fractures [15]. According to the latest report from American Academy of Orthopaedic Surgeons (AAOS), DHS was suggested for stable trochanteric fractures [16].

Following a systematic review, our aim was to conduct a meta-analysis first comparing the efficacy of PFN (including PFNA) and DHS for trochanteric fractures. Second, we wanted to summarize possible preventive solutions from a translational pre-clinical research perspective which might minimize the risk for implant-related complications in the future.

## Methods

### Protocol and registration

The protocol was registered (PROSPERO: CRD42021239974), following standard reporting methods [17]; which is available as Additional file 1: Appendix.

### Data sources and search strategy

An extensive electronic search for randomized trials was conducted by two independent investigators via three databases: PubMed, Web of Science and Cochrane Central Register. The last search was last updated on January 31, 2021. To identify the search terms, searches were performed using Medical Subject Headings (MeSH) combined with free words: “trochanteric femoral fracture”; “proximal femoral nail”; “dynamic hip screw”; and “randomized controlled trial”. The detailed search strategy for PubMed is as below:

((((“Femoral Fractures”[Mesh]) OR (“Hip Fractures”[Mesh])) OR ((((((((((((intertrochanteric fractures) OR (intertrochanteric fracture)) OR (trochanteric fractures)) OR (trochanteric fracture)) OR (pertrochanteric fractures)) OR (pertrochanteric fracture)) OR (Femoral intertrochanteric fracture)) OR (Femoral intertrochanteric fractures)) OR (intertrochanteric femoral fracture)) OR (intertrochanteric femoral fractures)) OR (IFFs)) OR (IFF))) AND (((((dynamic hip screw) OR (sliding hip screw)) OR (DHS)) OR (SHS)) OR (“Bone Screws”[Mesh]))) AND (((proximal femoral nail anti-rotation) OR (proximal femoral nail antirotation)) OR (PFNA)).

### Eligibility criteria

Randomized controlled trials (RCTs) comparing PFN with DHS for trochanteric fractures were included. The patients should be more than 18 years old with trochanteric fractures, defined as stable (AO/OTA 31-A1) or unstable fractures (AO/OTA 31-A2 and A3) [18]. Trials including participants with a history of significant trauma or systemic inflammatory conditions were not considered eligible.

### Study selection

Two independent investigators reviewed the studies. Inclusion criteria were: (1) a randomized controlled trial, (2) patients randomly assigned to DHS or PFN (incl. PFNA), (3) clinical data presented, including but not limited to operative time, intraoperative blood loss, intraoperative fluoroscopy time, achievement of closed reduction or postoperative complications like non-union, implant failure and revision surgery, (4) only English published studies were included. Any disagreement was resolved by discussion between the two investigators.

### Data collection process

The following data were extracted: year of publication, number of patients, characteristics of patients (age and sex), clinical outcomes including operative time, intraoperative blood loss, intraoperative fluoroscopy time, achievement of closed reduction, non-union, implant failure and revision surgery. Data extraction was done by reading the full article with interpretation of figures and tables in every study included.

### Risk of bias assessment

All studies were assessed for the risk of bias by referring to the Cochrane Handbook for Systematic Reviews of Interventions for the following domains: (1) random sequence generation (selection bias), (2) allocation concealment (selection bias), (3) blinding of participants and personnel (performance bias), (4) blinding of outcome assessment (detection bias), (5) incomplete outcome data (attrition bias), and (6) selective reporting (reporting bias). All risks of bias were evaluated with a grade of low, unclear, or high risk.

### Data synthesis and analysis

Mean Difference (MD) or Risk Ratio (RR) and 95% confidence interval (95%CI) were used as effect sizes depending on the measurement scale (Continuous [19] and Binary [20] outcomes, respectively). All statistical analyses were performed using RevMan software (version 5.3). We used visual inspection of the forest plots to investigate the possibility of statistical heterogeneity; this inspection was supplemented with, mainly, the I^2^ index, which describes the percentage of total variation across trials that is attributable to heterogeneity rather than to chance [21]. We used random-effects meta-analysis as the default option, while fixed effect models were applied for the purpose of sensitivity [22]. The stratified analysis according to PFN type, fracture pattern, during of the follow-up and origin of the study were also performed.

### Quality of evidence

After all the meta-analyses the quality of the evidence was evaluated based on the evidence profile using the GRADE (Grading of Recommendations Assessment, Development and Evaluation) system [23]. The GRADE approach enables a rating (down) of the overall quality based on the evidence for risk of bias, publication bias, imprecision, inconsistency, and indirectness. The GRADE ratings of very low-, low-, moderate-, or high-quality evidence reflect the extent to which we are confident that the effect estimates are correct.

## Results

### Study selection

**Fig. 1:**
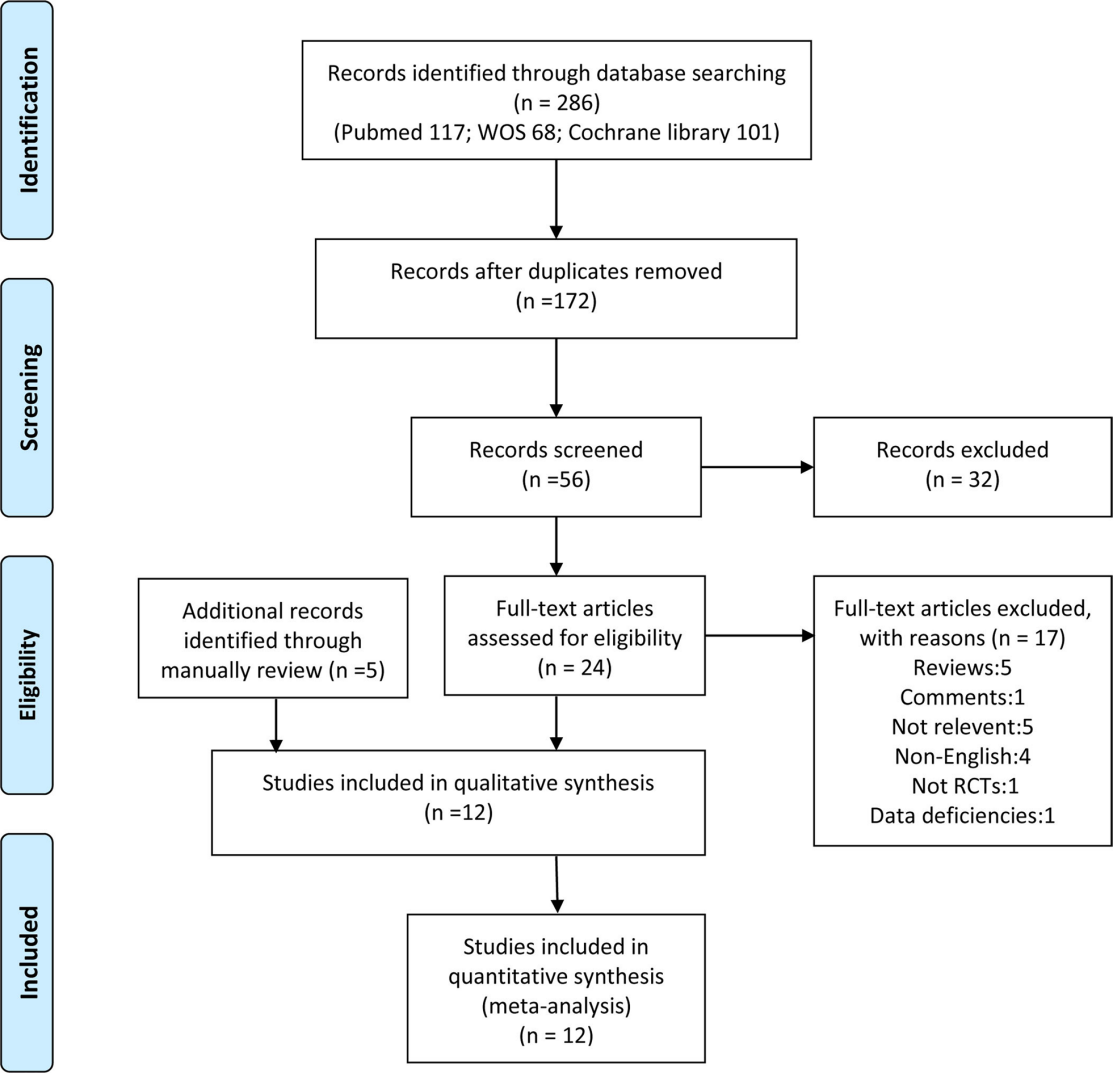
Flow diagram of the systematic literature search and selection of included studies.

Two hundred and eighty-six articles were yielded from database searches. One hundred and fourteen duplications were removed due to duplicates. One hundred and sixteen were dropped after viewing the abstract. Thirty-two studies were removed after full-text assessment. Only 7 articles met the primary inclusion criteria which were taken for this meta-analysis. Meanwhile, we manually reviewed the reference lists of relevant reviews to identify any eligible studies meeting our inclusion criteria, which added another 5 RCTs. The selection process was shown in PRISMA Flow Diagram (Fig. 1).

### Study characteristics

The baseline characteristics of each included study are presented in Table 1. The identified 12 RCTs [6, 12, 18, 24–32] comparing PFN(A) with DHS included 1889 trochanteric fracture patients, with 934 patients allocated to PFN(A) and 955 for DHS. Four RCTs [6, 25, 28, 29] only included patients with stable fractures while 5 studies [12, 24, 26, 29, 31] only included unstable fractures. Another 3 RCTs [18, 27, 32] included both stable and unstable fractures as mixed patients. The shortest follow-up was 4 months and the longest as 48 months. Most studies had a follow-up at least for one year.

**Table 1:** Characteristics of the included studies.

Study	Fracture (stable/unstable/Mix)	Follow-up (months)	Number of patients (n)		Average age (Years)		Sex (M/F)	
			PFN(A)	DHS	PFN(A)	DHS	PFN(A)	DHS
Adeel K, 2020	Unstable	12	34	34	59.23	60.88	25/9	22/12
Huang SG, 2017	Unstable	12	30	30	75.07	74.01	15/30	17/13
Pajarinen J, 2005	Stable	4	54	54	80.9	80.3	13/41	14/40
Papasimos S, 2005	Unstable	12	40	40	79.4	81.4	17/23	14/26
Parker MJ, 2012	Mix	12	300	300	82.4	81.4	52/248	69/231
Saudan M, 2002	Mix	12	100	106	83.0	80.7	24/76	22/84
Sharma A, 2018	Stable	24	31	29	60.67	62.27	19/12	19/10
Singh NK, 2019	Stable	12	30	30	77.76	69.33	9/21	16/14
Xu YZ, 2010	Unstable	12	51	55	78.5	77.9	15/36	16/39
Yu WG, 2016	Stable	48	110	112	72.02	73.05	51/59	57/55
Zehir S, 2015	Unstable	6	96	102	77.22	76.86	37/59	39/63
Zou J, 2009	Mix	12	58	63	65.0	65.0	12/46	15/48

### Risk of bias

**Fig. 2:**
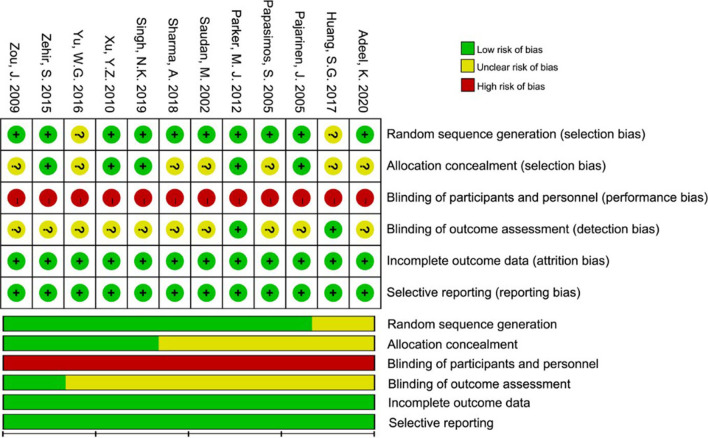
Risk of bias of included trials.

Random sequence generation was reported in all RCTs, although 2 RCTs [24, 30] did not disclose the detailed method. Sealed envelope technique, which was regarded as a random method for allocation of the patients, was employed in 5 studies [25, 27–29, 31]. Blinding of participants who performed the operation is almost impractical, so the performance bias was marked high risk in every study. Detection bias (blinding of outcome assessment) was marked low risks in only 2 RCTs [24, 27]. Attrition bias and reporting bias were low in all RCTs. A review of the authors’ judgment about the risk of bias is shown in Fig. 2.

### Intraoperative clinical outcomes

Eleven studies [6, 12, 18, 24–29, 31, 32] reported the operative time indicating PFN(A) had a shorter operative time than DHS with an overall effect size of -9.49 min (95% CI, − 18.74 to − 0.25) (Additional file 2: Fig. S1). The stratified analysis showed PFNA, instead of PFN, is the main reason for shorter operative time [effect size (ES), − 17.7; 95% CI, − 32.6 to − 2.8] (Table 2). No difference was seen in different type of fractures (Stable/Unstable/Mix) when comparing PFN(A) with DHS (Table 2). The studies which aimed for short follow-up (less than 6 months) seems to have less effect on operative time between PFN(A) and DHS compared to the studies with intermediate or long follow-up (Table 2). Interesting result about different countries is that Asian countries like China, India and Pakistan tend to report shorter operative time for PFN(A) compared to western countries (Finland, Greece, Switzerland, and UK) (Table 2). The risk of bias for stratified analysis of operative time is also shown in Table 2. Eight RCTs [6, 12, 24, 25, 28, 29, 31, 32] reported the intraoperative blood loss. The result showed that PFN(A) was associated with less amount of intraoperative blood loss compared to DHS with an overall effect size of − 158.2 mL (95% CI, − 203.05 to − 113.34). Both PFN and PFNA had less intraoperative blood loss compared to DHS with an effect size of − 136.29 mL and − 177.35 mL, respectively (Additional file 3: Fig. S2). The data from 7 RCTs [18, 26–29, 31, 32] also showed that PFN(A) needed more intraoperative fluoroscopy duration as guidance compared to DHS with an overall effect size of 0.43 min (95% CI, 0.18 to 0.68). Compared to DHS, PFNA need more intraoperative fluoroscopy duration (MD, 0.73; 95% CI, 0.19 to 1.26) than PFN (MD, 0.16; 95% CI, 0.01 to 0.32) (Additional file 4: Fig. S3). Five RCTs [12, 18, 24, 25, 27] reported the proportion of CRIF while 2 [24, 25] of them reported all the operations being successfully completed with close reduction. No difference was seen for achievement of closed reduction between PFN and DHS (RR, 1.02; 95% CI, 0.99 to 1.05) (Additional file 5: Fig. S4).

**Table 2:** Results of the stratified meta-analyses for operative time.

Variable	Trials (no.)	ES	95% CI		Tau^3	I^2 (modified)
All trials	12	− 9.4	− 19.98	1.18	311.9(*)	
Intervention					280.8	
PFN	6	− 2.4	− 16.32	11.52	90%	88%
PFNA	5	− 17.7	− 32.6	− 2.8		
Fracture pattern					383.1	
Stable	4	− 6.2	− 28.94	16.54	123%	120%
Unstable	5	− 900	− 26.44	8.44		
Mix	3	− 13.1	− 35.44	9.24		
Follow-up					372.4	
Short	2	− 1.5	− 28.55	25.55	119%	117%
Intermediate	8	− 11,00	− 24.72	2.72		
Long	2	− 12.8	− 52,00	26.4		
Country					532.14	
China	4	− 20,00	− 46.26	6.26	171%	167%
Finland	1	10,00	− 35.86	55.86		
Greece	1	12,00	− 35.63	59.63		
India	2	− 14.6	− 47.33	18.13		
Pakistan	1	− 23.4	− 68.68	21.88		
Switzerland	1	− 100	− 46.86	44.86		
Turkey	1	− 12.5	− 57.78	32.78		
UK	1	300	− 42.28	48.28		
Random sequence generation					293.4	
Low	10	− 7.3	− 18.28	3.68	94%	92%
High	0					
Unclear	2	− 30.7	− 64.8	3.4		
Allocation concealment					256	
Low	5	− 0.6	− 14.91	13.71	82%	80%
High	0					
Unclear	7	− 17.1	− 30.43	− 3.77		
Blinding of outcome assessment					344.5	
Low	2	− 13.6	− 39.47	12.27	110%	108%
High	0					
Unclear	10	− 8.4	− 20.75	3.95		
Incomplete outcome data					311.9	
Low	12	− 9.42	− 20,00	1.16	100%	98%
High	0					
Unclear	0					
Selective reporting					311.9	
Low	12	− 9.42	− 20,00	1.16	100%	98%
High	0					
Unclear	0					

* means *p* < 0.05; Caps () were used for annotating

Abbreviations: *ES* effect size, *CI* confidence interval

### Postoperative complications

No difference in postoperative complications was seen for non-union between PFN(A) and DHS (1.7% vs. 2%; RR, 0.93; 95% CI, 0.44 to 1.96) (Additional file 6: Fig. S5). Ten RCTs [6, 12, 18, 24, 25, 27, 29–32] reported implant failure in PFN(A) and DHS, with no significant difference (2.5% vs. 3.5%; RR, 0.78; 95% CI, 0.35 to 1.75) (Additional file 7: Fig. S6). Four RCTs [18, 26–28] reported the number of patients under revision. In PFN(A), 2.2% patients needed further revision surgery, while 2.9% for DHS. There was no difference between the two methods with an overall effect size of 0.84 (95% CI, 0.22 to 3.15) (Additional file 8: Fig. S7).

### Quality of evidence for each outcome

Overall evidence was qualified using GRADE for included RCTs evaluating each outcome (Table 3). Serious risk of bias was qualified Yes due to the high risk of bias for blinding of participants and personnel for all studies. Inconsistence (*I*^2^), indicating heterogeneity between studies, was marked as Yes for operative time, blood loss and intraoperative fluoroscopy, with *I*^2^ > 95%. While it was No for closed reduction, non-union, implant failure and revision, with *I*^2^ < 55%. No serious indirectness was seen for all outcomes. Serious impression was only qualified Yes for operative time and intraoperative fluoroscopy time based on the effect size, 95% CI and clinical significance. At the end, we could see that the evidence for the difference in operative time is very low and low for intraoperative blood loss and fluoroscopy time. For the other 4 outcomes (closed reduction, non-union, implant failure and revision surgery), the evidence level is moderate.

**Table 3:** GRADE evidence profile of PFN versus DHS for patients with trochanteric fracture included in randomized clinical trials.

Outcome	Number of trials (≤ 12 total)	No. of patients (*N* ≤ 1889 total)	Follow-up, months (min; max)	Serious risk of bias?	Inconsistency: I^2^ (%)	Serious indirectness?	Serious imprecision?	Serious risk of reporting bias?	Effect size (95%CI)	Quality of the evidence
Operative time	11	1667	Intraoperation	Yes	Yes(98%)	No	Yes	No	− 9.49(− 18.74 to 0.25)	Very low
Blood loss	8	781	Intraoperation	Yes	Yes (95%)	No	No	No	− 158.20 (− 203.05 to − 113.34)	Low
Fluoroscopy time	7	1371	Intraoperation	Yes	Yes (99%)	No	Yes	No	0.43 (0.18 to 0.68)	Low
Closed reduction	5	1042	Post-operation	Yes	No (52%)	No	No	No	1.02 (0.99 to 1.05)	Moderate
Non-Union	5	1091	12;12	Yes	No (0%)	No	No	No	0.93(0.44 to 1.96)	Moderate
Implant Failure	10	1749	4;24	Yes	No (32%)	No	No	No	0.78 (0.35 to 1.75))	Moderate
Revision	4	886	12;12	Yes	No (44%)	No	No	No	0.84 (0.22 to 3.15)	Moderate

## Discussion

Dynamic hip screw used to be the gold standard of trochanteric fracture treatment, especially for the stable fractures [6, 33]. PFN(A) is a newer implant, which consists of a funnel-shaped intramedullary nail with slight bending to reflect proximal femoral diaphyseal trochanteric morphology. The main advantage of PFN(A) is to reduce surgical trauma inflicted to bone and soft tissue [24, 34]. However, which technique is more suitable for trochanteric hip fractures is still controversial. Present study revealed that PFN(A) had a shorter operative time and less intraoperative blood loss, but required more intraoperative fluoroscopy time compared to DHS. No difference was seen for post-operative complications like implant failure, non-union and revision surgery.

For PFN(A), a shorter operative time was achieved compared to DHS, especially for PFNA. However, we did not see a difference between stable or unstable fracture due to the small sample size in each subgroup. The same trend was seen for intraoperative blood loss that PFN(A) had less blood loss compared to DHS. The shorter operative time and less blood loss might be due to the smaller incision and reduced muscle injury. The PFN implant is also placed through a minimally invasive approach without opening the fracture site, while DHS requires a larger incision [24, 34].

For intraoperative fluoroscopy, DHS had less exposure compared to PFN(A). Since PFN(A) is done through a minimally invasive approach, it can be expected that more fluoroscopy guidance was needed to confirm the proper implant placement with good stability and less implant failure. Thus, DHS might be a choice for those patients who could bear limited radiation dose or with multiple chronic diseases. Surgical staff’s exposure to radiation must be taken into consideration especially for developing countries.

A closed reduction is defined as a procedure to line up the ends of a fracture by manipulation of bone fragments without surgical exposure of tissues surrounding the fragments. In our study, proportion of successful closed reduction was similar in PFN(A) and DHS (PFN 98.8% and DHS 95%, respectively). More clinical studies need to be conducted to explore the effect of PFN(A) and DHS on successful closed reduction for stable or unstable trochanteric fractures.

The incidence of postoperative complications, including non-union of fracture, implant failure, revision of fixation failure or arthroplasty, was not significantly different between PFN and DHS. Non-union and implant failure are the common complications directly related with compromised fixation stability [35]. In the current meta-analysis, the overall ratios for non-union and implant failure in PFN were 1.9% and 2.2%. For DHS, it was 2% and 3.5%, respectively. It is reasonable to assume that these two complications contributed to a revision rate of 2.4% and 2.9% in PFN and DHS, respectively, where patients received secondary fixation or arthroplasty. To combat such failures, different internal fixation implants with specifically designed mechanical properties are being developed. The proximal femoral nail antirotation device (PFNA) was designed, with a smaller distal shaft diameter, resulting in a lower concentration of stress in the tip than in the PFN. The helical neck blade in the PFNA prevents the bone damage that occurs during drilling and insertion of the standard sliding hip screw [36–38] by radial compaction of the cancellous bone during insertion [29]. However, it still was not associated with decreased postoperative complications compared to DHS. For non-union, PFNA had a ration of 1.2% compared to DHS with 2%. Moreover, 2.9% of patients underwent PFNA had implant failure compared to DHS with 3.5%. In our study, evidence pointed out that postoperative implant related complications, like screw dislocation or cut-out, has not been improved by modifying the surgical technique. Previous studies have shown that PFN(A) device, irrespective of the brand, had similar incidence in terms of post-operative complications [13, 39]. Furthermore, it was also found that the more expensive device is not positive related to the better short-term outcomes [40]. This indeed demands the development of new technologies with further exploration, and we believe clues from the pre-clinical research with high translational potential may help decrease these risks.

Although research on developing implant designs for fixation of hip fractures is promising, augmenting bone implant interface utilizing absorbable and non-absorbable materials between metal implants and osteoporotic bone are emerging as the future direction [41]. Resorbable ceramic cement was reported to increase the implant fixation and prevent excessive screw sliding and cutout [42, 43]. Recently, Joeri Kok et al. have confirmed an injectable biphasic bone substitute could theoretically increase the initial hip cancellous fracture strength [44]. Same team also developed a new device that allows biomaterial injection trough the hip screw during surgery which, when combined with controlled delivery of bone active molecules, could increase bone formation around the screw threads for a more stable fixation [45]. Furthermore, it was recently shown that it is possible to recruit bisphosphonates like zoledronic acid and biomodulate the hydroxyapatite particles to get more bone formation for better screw anchorage [46, 47]. Translation of these augmentation methods into clinical trials and practice in the future has been suggested to be feasible with appropriate stratification of patients [48, 49].

## Limitations

Our study was limited in several aspects. First, available articles were still not sufficient, especially RCTs of stable trochanteric fractures. Second, some studies were not appropriate because of the small sample size, half of the included studies were small sample sized RCTs with less than 100 patients. Lastly, Patient reported outcomes such as pain and activities of daily living (ADL) were not extracted.

## Conclusion

PFN(A) results in a significantly shorter operation time and less intraoperative blood loss compared to DHS. However, it did not significantly decrease the risk of post-operative complications like implant cut-out and screw sliding. In the future, novel solutions for improving bone anchorage and reducing the risk of implant failure need to be explored further.

## Supplementary Information

**Additional file 1: Appendix. **PROSPERO registration

**Additional file 2: Fig. S2. **Comparing operative time between PFN(A) and DHS.

**Additional file 3: Fig. S3. **Comparing intraoperative blood loss between PFN(A) DHS.

**Additional file 4: Fig. S4. **Comparing intraoperative fluoroscopy time between PFN(A) and DHS.

**Additional file 5: Fig. S5. **Comparing closed reduction between PFN (A) and DHS.

**Additional file 6: Fig. S6. **Comparing post-operative non-union between PFN(A) and DHS.

**Additional file 7: Fig. S7. **Comparing postoperative implant failure between PFN(A) and DHS.

**Additional file 8: Fig. S8. **Comparing revision surgery between PFN(A) and DHS.

## Data Availability

The data used to support the findings of this study are included within the articles.
