# Supplementary material for: Comparative effectiveness research on proximal femoral nail versus dynamic hip screw in patients with trochanteric fractures: a systematic review and meta-analysis of randomized trials
Source: J Orthop Surg Res. 2022 Jun 3;17:292. doi: 10.1186/s13018-022-03189-z (PMC9164432; doi:10.1186/s13018-022-03189-z)
Supplement: Supplementary file 1 — Additional file 1: Appendix. PROSPERO registration [file 13018_2022_3189_MOESM1_ESM.docx]

Appendix 1 PROSPERO registration

Appendix 1
