# Supplementary figures and images for: Comparative effectiveness research on proximal femoral nail versus dynamic hip screw in patients with trochanteric fractures: a systematic review and meta-analysis of randomized trials
Source: J Orthop Surg Res. 2022 Jun 3;17:292. doi: 10.1186/s13018-022-03189-z (PMC9164432; doi:10.1186/s13018-022-03189-z)

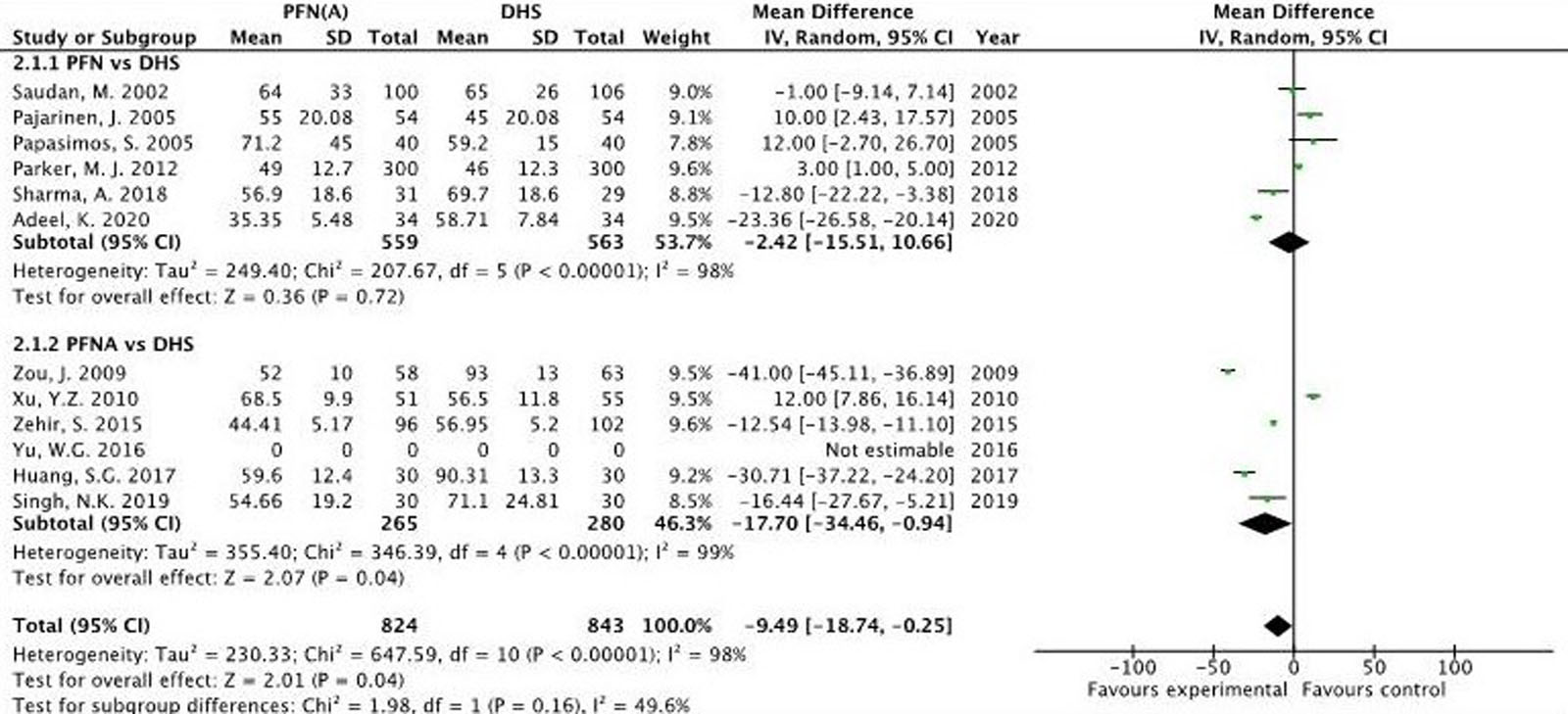

Supplement: Supplementary file 2 — Additional file 2: Fig. S2. Comparing operative time between PFN(A) and DHS. [file 13018_2022_3189_MOESM2_ESM.jpg]

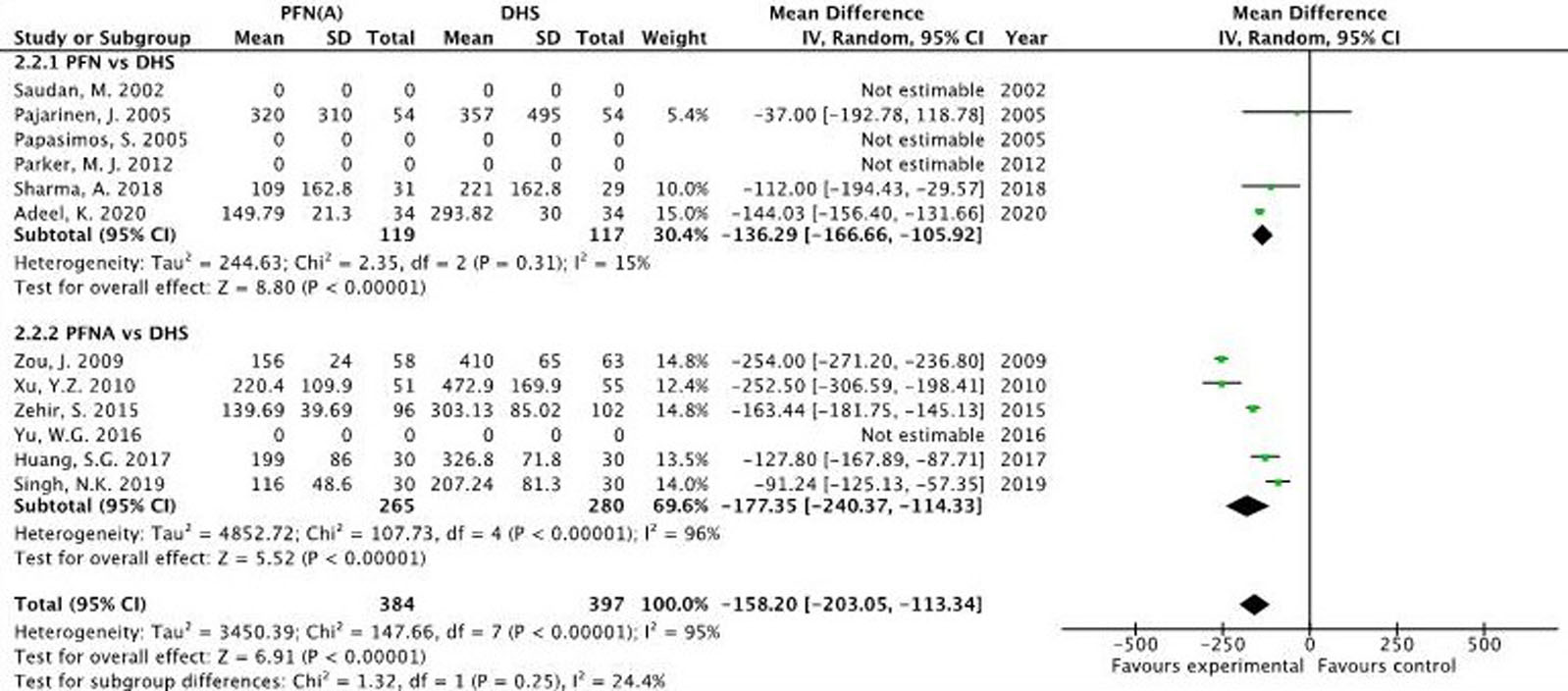

Supplement: Supplementary file 3 — Additional file 3: Fig. S3. Comparing intraoperative blood loss between PFN(A) DHS. [file 13018_2022_3189_MOESM3_ESM.jpg]

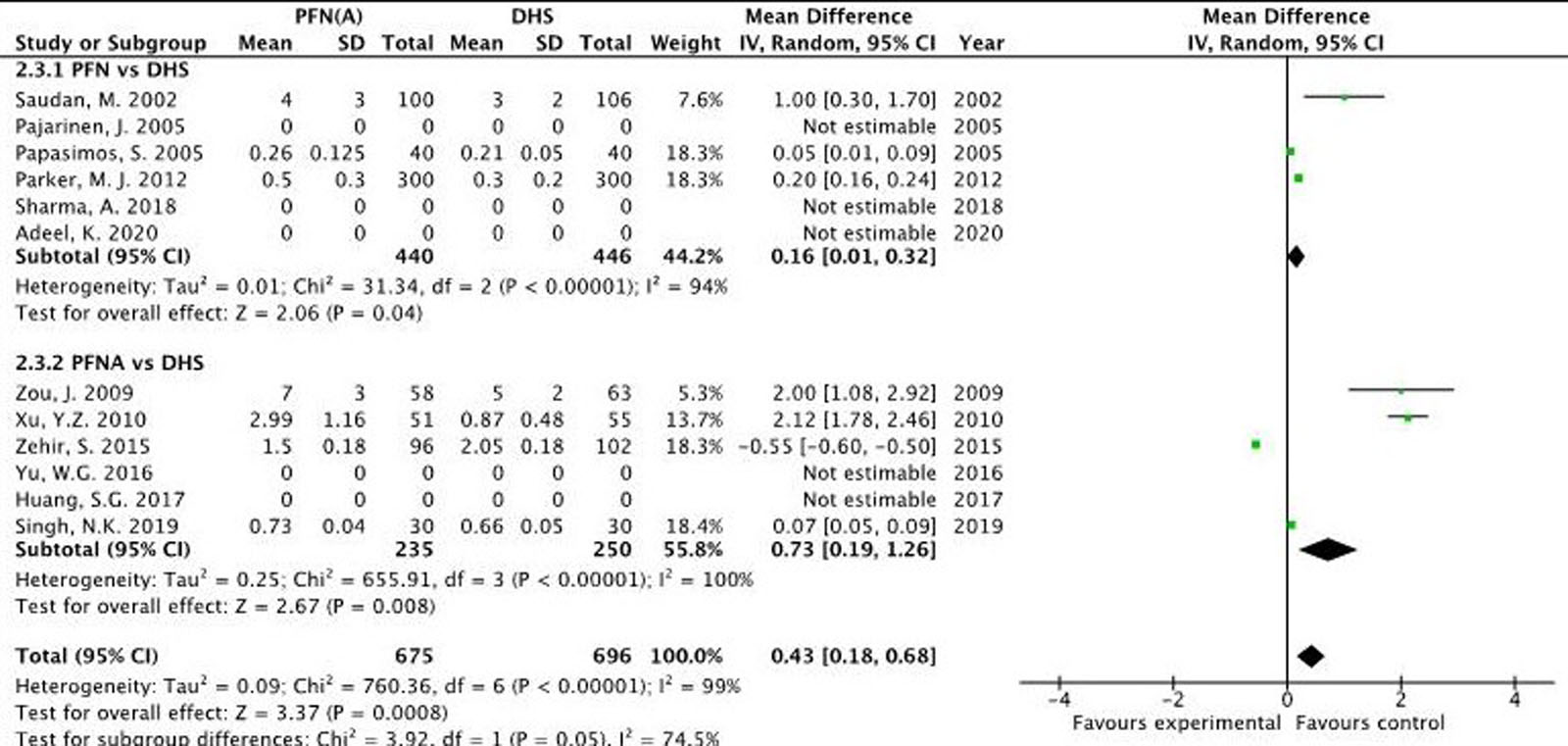

Supplement: Supplementary file 4 — Additional file 4: Fig. S4. Comparing intraoperative fluoroscopy time between PFN(A) and DHS. [file 13018_2022_3189_MOESM4_ESM.jpg]

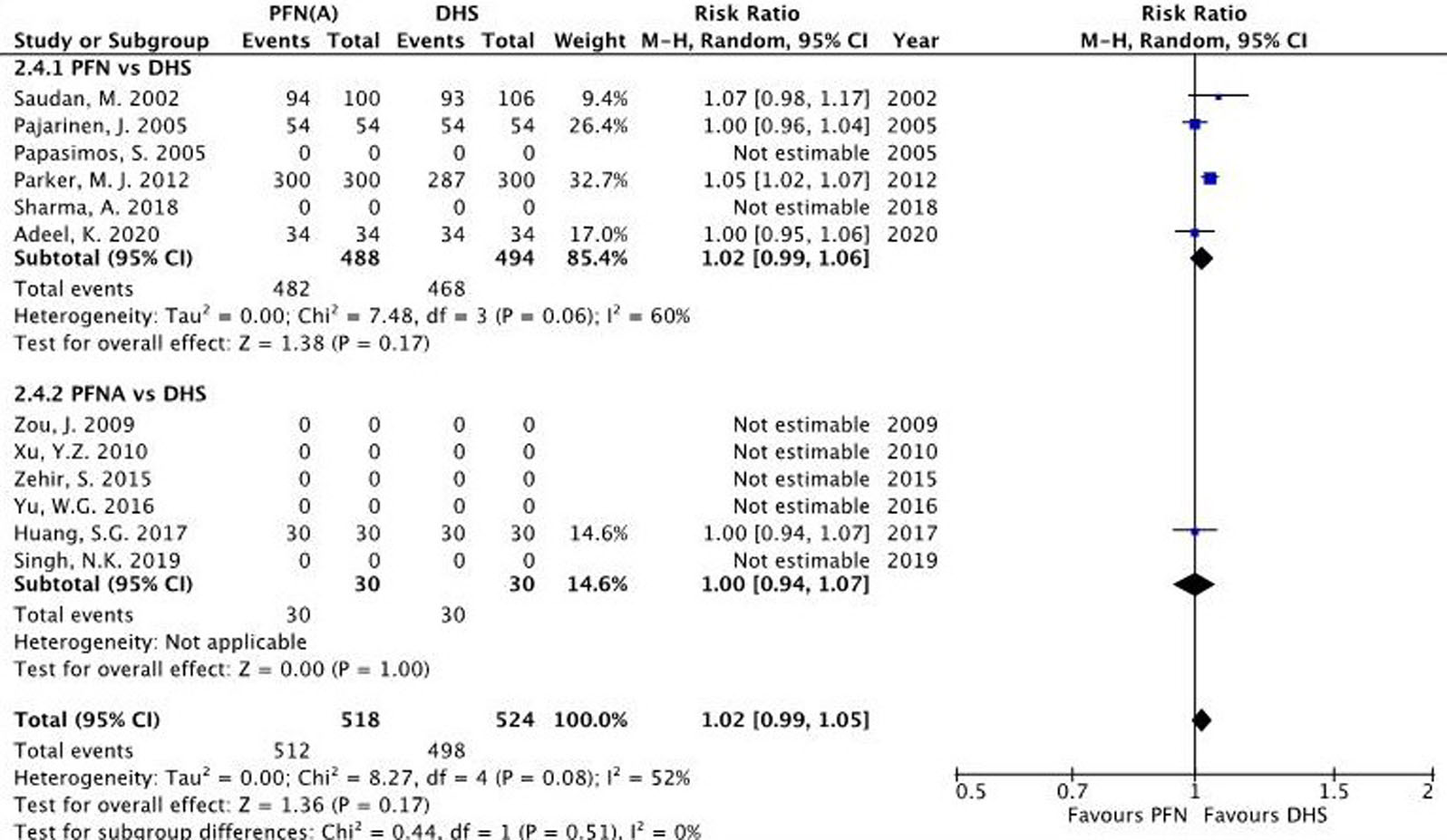

Supplement: Supplementary file 5 — Additional file 5: Fig. S5. Comparing closed reduction between PFN (A) and DHS. [file 13018_2022_3189_MOESM5_ESM.jpg]

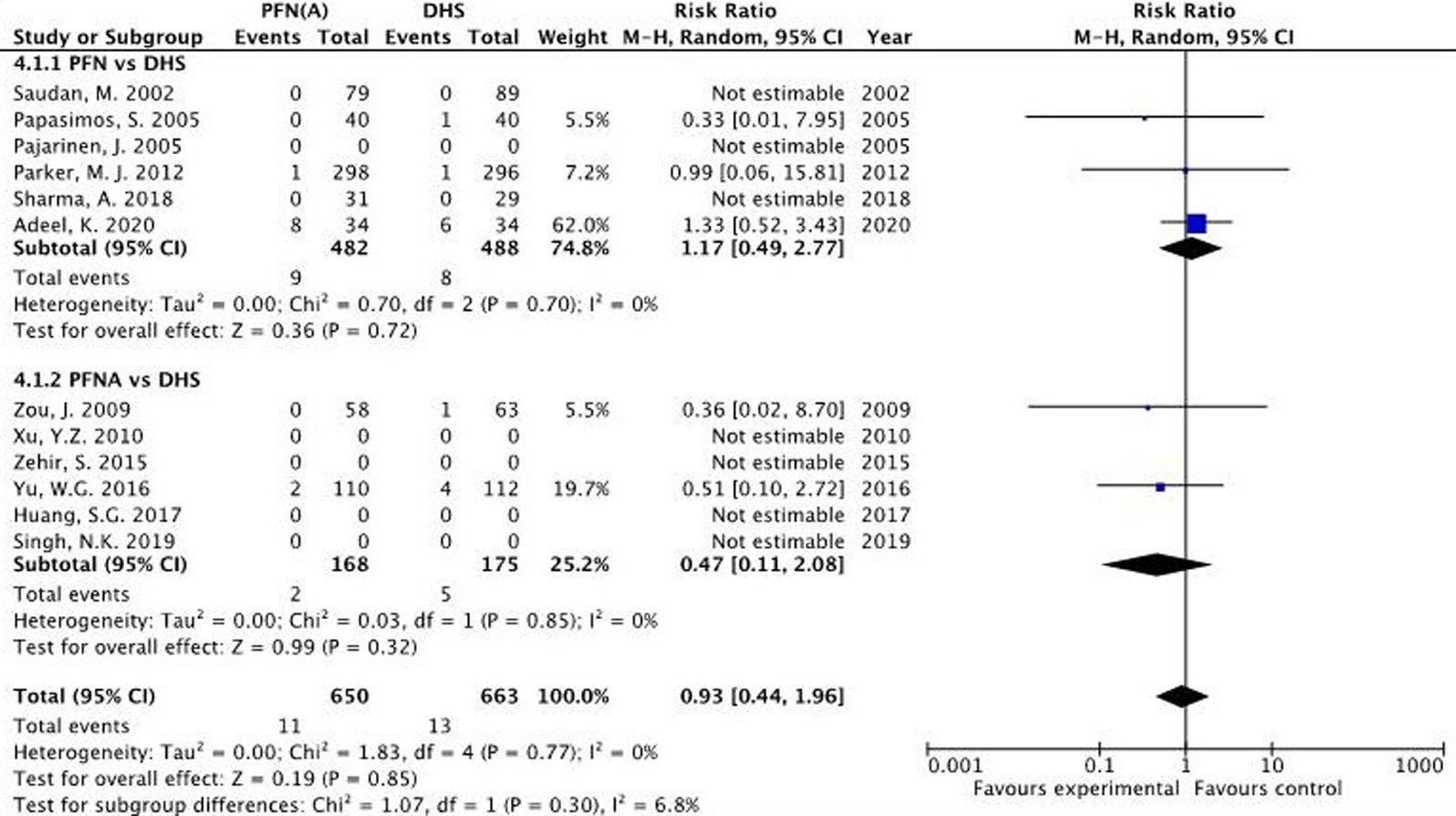

Supplement: Supplementary file 6 — Additional file 6: Fig. S6. Comparing post-operative non-union between PFN(A) and DHS. [file 13018_2022_3189_MOESM6_ESM.jpg]

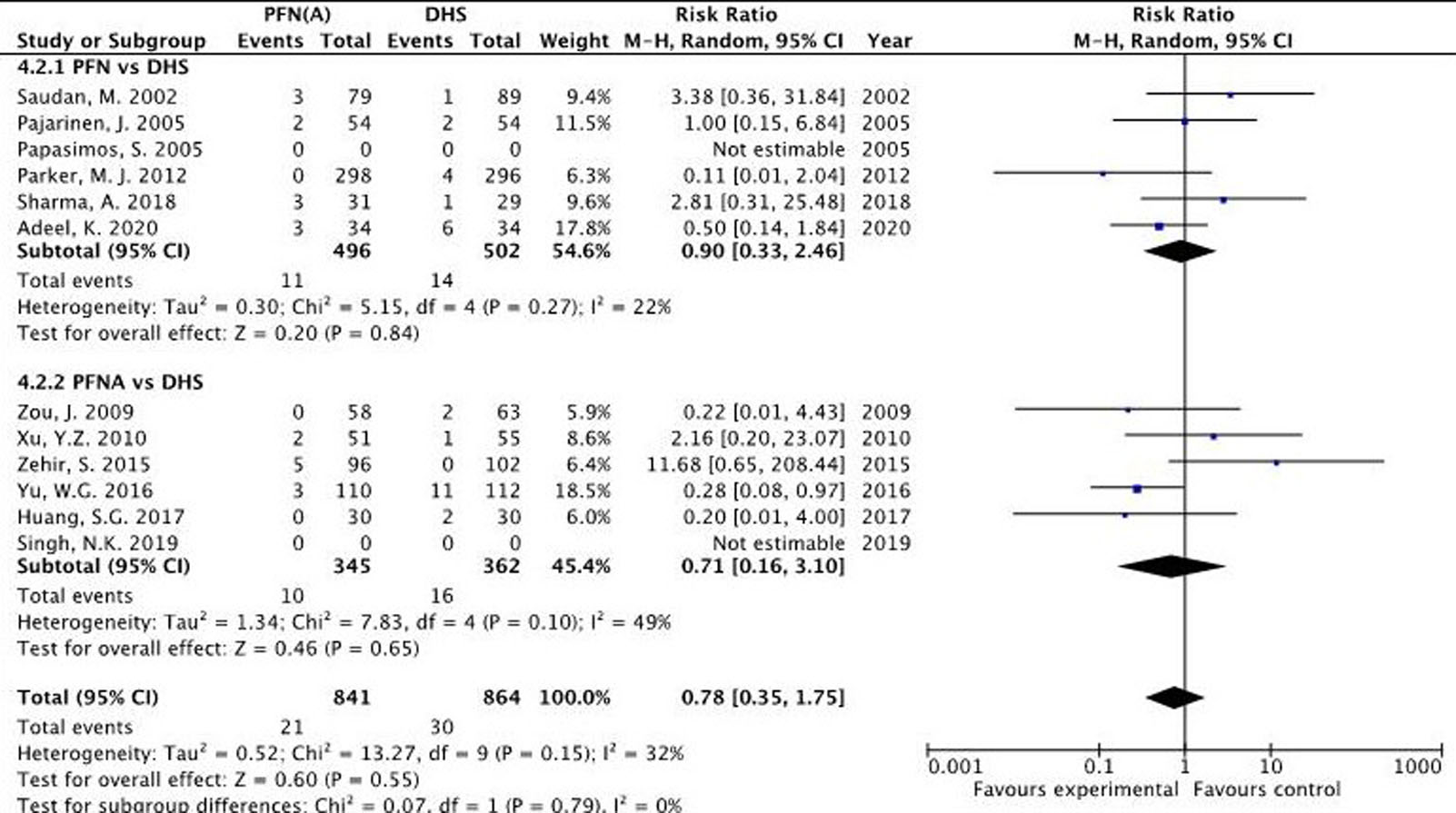

Supplement: Supplementary file 7 — Additional file 7: Fig. S7. Comparing postoperative implant failure between PFN(A) and DHS. [file 13018_2022_3189_MOESM7_ESM.jpg]

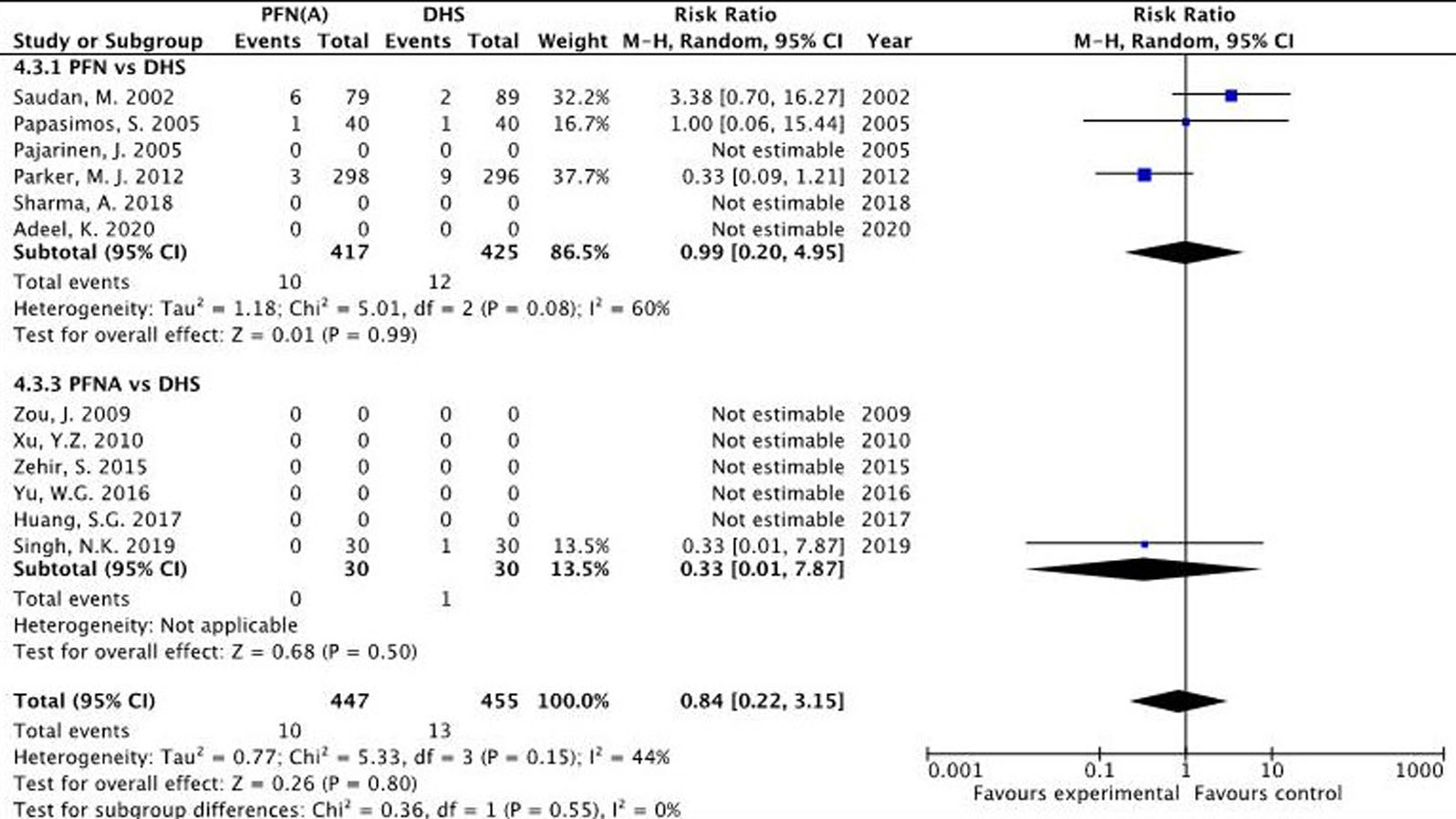

Supplement: Supplementary file 8 — Additional file 8: Fig. S8. Comparing revision surgery between PFN(A) and DHS. [file 13018_2022_3189_MOESM8_ESM.jpg]
